# Phytotoxicity of Four Photosystem II Herbicides to Tropical Seagrasses

**DOI:** 10.1371/journal.pone.0075798

**Published:** 2013-09-30

**Authors:** Florita Flores, Catherine J. Collier, Philip Mercurio, Andrew P. Negri

**Affiliations:** 1 Australian Institute of Marine Science, Townsville, Queensland, Australia; 2 School of Marine and Tropical Biology, James Cook University, Townsville, Queensland, Australia; 3 School of Medicine, University of Queensland and National Research Centre for Environmental Toxicology, Coopers Plains, Queensland, Australia; University of Hyderabad, India

## Abstract

Coastal waters of the Great Barrier Reef (GBR) are contaminated with agricultural pesticides, including the photosystem II (PSII) herbicides which are the most frequently detected at the highest concentrations. Designed to control weeds, these herbicides are equally potent towards non-target marine species, and the close proximity of seagrass meadows to flood plumes has raised concerns that seagrasses may be the species most threatened by herbicides from runoff. While previous work has identified effects of PSII herbicides on the photophysiology, growth and mortality in seagrass, there is little comparative quantitative toxicity data for seagrass. Here we applied standard ecotoxicology protocols to quantify the concentrations of four priority PSII herbicides that inhibit photochemistry by 10, 20 and 50% (IC_10_, IC_20_ and IC_50_) over 72 h in two common seagrass species from the GBR lagoon. The photosystems of seagrasses *Zostera* *muelleri* and *Halodule* *uninervis* were shown to be generally more sensitive to the PSII herbicides Diuron, Atrazine, Hexazinone and Tebuthiuron than corals and tropical microalgae. The herbicides caused rapid inhibition of effective quantum yield (*∆F/F*_*m*_ *′*), indicating reduced photosynthesis and maximum effective yields (*F_v_/F_m_*) corresponding to chronic damage to PSII. The PSII herbicide concentrations which affected photosynthesis have been exceeded in the GBR lagoon and all of the herbicides inhibited photosynthesis at concentrations lower than current marine park guidelines. There is a strong likelihood that the impacts of light limitation from flood plumes and reduced photosynthesis from PSII herbicides exported in the same waters would combine to affect seagrass productivity. Given that PSII herbicides have been demonstrated to affect seagrass at environmental concentrations, we suggest that revision of environmental guidelines and further efforts to reduce PSII herbicide concentrations in floodwaters may both help protect seagrass meadows of the GBR from further decline.

## Introduction

### Pesticide contamination in the tropics

The lagoon of the World Heritage listed Great Barrier Reef (GBR) is contaminated with a range of agricultural pesticides including herbicides and insecticides [1]. Concentrations of these pesticides are highest nearshore and during the summer wet season (November to March) when high rainfall facilitates transport from farms, through the catchments and into the lagoon [2–4]. The most frequently detected pesticides are the photosystem II (PSII) herbicides such as Diuron, Atrazine, Hexazinone and Tebuthiuron [2,3,5,6], and recent modelling indicates that over 15,000 kg of PSII herbicides alone enter the GBR lagoon along its 2,600 km shoreline on an annual basis [7–9]. Although the GBR and its catchments are the most heavily monitored of all tropical ecosystems for pesticides, the issue is not restricted to Australia. Similar herbicides are considered a potential threat to nearshore habitats of the western Indian Ocean [10], the northern Pacific [11], the Atlantic coast, including Chesapeake Bay [12] and the Caribbean [13].

### Effects of PSII herbicides on non-target marine species

PSII herbicides act by binding to the D1 protein in PSII which blocks photosynthetic electron flow and this in turn limits the fixation of CO_2_ in plants [14]. Under moderate light conditions PSII herbicides reduce primary productivity, and under higher light, blockage of the electron transport system results in the build-up of reactive oxygen species that damage PSII [15,16]. These herbicides have been designed to prevent germination, reduce growth and kill weeds, and given that the D1 protein is one of the most highly conserved proteins across taxa, it is not surprising that PSII herbicides also affect non-target marine species at low concentrations [17]. Since photosynthesis fixes carbon for growth, the effects of PSII herbicides on photosynthesis result in reduced primary production which can have flow-on effects at higher trophic levels in marine ecosystems [18].

The sensitivities of several tropical marine taxa to PSII herbicides have been tested in controlled laboratory conditions. The non-invasive technique of pulse amplitude modulation (PAM) fluorometry (see Methods section) is particularly suited to quantify the sub-lethal effects of PSII herbicides on plants as the parameters measured are directly linked to reduced photochemical efficiency and/or capacity by binding of the herbicide in PSII [16,18]. PAM fluorometry has been used to measure the direct effects of PSII herbicides on photosynthetic efficiency and damage to photosystem II in corals [19–22], microalgae [23–25], Foraminifera [26] and crustose coralline algae [19], providing regulators and managers with growing toxicity datasets for herbicide and species comparisons.

### Seagrass and herbicides

Seagrass meadows were identified as being at risk from Diuron and/or Atrazine exposure more than three decades ago off the US [27,28] and European [29] coasts and more recently within the GBR lagoon [1]. Diuron was detected within the leaf, root and rhizome tissue of seagrass at 1.1 µg kg^-1^ from Cardwell (GBR) and 1.7 µg kg^-1^ in seagrasses from Moreton Bay, just south of the GBR [1]. A wider range of PSII herbicides including Simazine, Hexazinone, Ametryn and Tebuthiuron were also detected in sediments of seagrass meadows and surface waters in the southern GBR lagoon [30]. A series of publications have reported that seagrasses are very sensitive to PSII herbicides, particularly Diuron and Atrazine, with inhibition of photosynthetic efficiency (*ΔF/F*’_*m*_) measured by PAM fluorometry the most commonly used endpoint (Table 1). Ralph [31] demonstrated inhibition of *ΔF/F*’_*m*_ in *Halophila* *ovalis* at Diuron and Atrazine concentrations as low as 10 µg l^-1^ (but lower concentrations were not tested). Haynes et al. [32] observed significant effects of Diuron on three seagrass species in aquaria over 5 days at similar concentrations and this was followed by recovery of *ΔF/F*’_*m*_ in most treatments (Table 1). Reductions in seagrass growth has also been measured over 4 weeks under low light conditions at 10 µg l^-1^ Diuron and reductions in total chlorophyll and mortality at 100 µg l^-1^ Diuron [33]. Other endpoints such as oxygen production have been measured on largely temperate species (reviewed in 34). However, impairment of photosynthetic processes (*ΔF/F*’_*m*_ and *F_v_/F*_*m*_) have been the most rapid and sensitive endpoints tested with the lowest significant effect concentration reported as 1 µg l^-1^ [10,35].

**Table 1:** Effect concentrations of agricultural PSII herbicides to seagrass in previous laboratory exposure experiments.

**Herbicide**	**Seagrass species**	**Endpoint**	**Duration**	**LOEC**	**IC_50_**	**Reference**
Diuron	* Halophila ovalis *	*ΔF/F’_m_*	5 & 72 h	10^a^	-	[31]
	* Halophila ovalis *	*ΔF/F’_m_*	5 d	10^b^	-	[32]
	* Zostera muelleri *	*ΔF/F’_m_*	5 d	10^b^	-	[32]
	* Zostera muelleri *	*ΔF/F’_m_*	96 h	10^a,c^		[69]
	* Zostera marina *	*F_v_/F_m_*	10 d	1^e^	3.2^e^	[35]
	* Zostera marina *	*Growth*	10 d	5^e^	-	[35]
	* Cymodocea serrulata *	*ΔF/F’_m_*	5 d	10^b^	-	[32]
	* Thalassodendron ciliatum *	*F_v_/F_m_*	72 h	1^c^	7.9^c,d,e^	[10]
Atrazine	* Halophila ovalis *	*ΔF/F’_m_*	5 & 72 h	10^a^	-	[31]
	* Zostera muelleri *	*ΔF/F’_m_*	4 h	10^a,c^	-	[69]
	* Zostera marina *	*ΔF/F’_m_ & F_v_/F_m_*	24 h	4^a,e^	-	[33]
	* Zostera marina *	Growth	21 d	100		[59]
	* Zostera marina *	Mortality	21 d	100		[59]
	* Zostera marina *	Growth	10 d	1900^e^	-	[12]
	* Zostera marina (seedlings)*	*ΔF/F’_m_ & F_v_/F_m_*	24 h	2^a,e^	-	[33]
	* Zostera marina (seedlings)*	Growth	4 wks	10^e^	-	[33]
	* Zostera marina (seedlings)*	Chlorophyll	4 wks	100^e^	-	[33]
	* Zostera marina (seedlings)*	Mortality	4 wks	100^e^	-	[33]
Simazine	* Halophila ovalis *	*ΔF/F’_m_*	5 & 72 h	100	-	[31]

LOEC is the lowest observed effect concentration (µg l^-1^); IC_50_ is the concentration (µg l^-1^) that inhibits 50% photosynthetic capacity. Inhibition of the effective (*ΔF/F*’_*m*_) and maximum (*F_v_/F*_*m*_) quantum yields from PAM fluorometry represent impairment of photosynthetic activity (see Methods section).

a lower concentrations not tested

b likely effects at lower concentrations but large uncertainties, temperature range 20-35°C

c rapid recovery in uncontaminated water

d estimated from 3 Diuron concentrations

e plants exposed without sediments

### Ecological threats of herbicides to seagrass

Coastal seagrass meadows are among the most ecologically important (and threatened) habitats in the tropics, providing critical ecosystem services including food for fish, turtle, manatee and dugong, habitat for fish and invertebrates and they are highly valued for their role in nutrient cycling [36,37]. Coastal communities across the globe are in turn dependent on the ecosystem services provided by seagrass meadows [38], and seagrass meadows enhance the ecosystem services of adjacent habitats such as coral reefs [39]. Recent estimates indicate global seagrass losses of 110 km^2^ yr^−1^ are comparable to those of tropical rainforests and coral reefs [40] and are primarily due to human impacts in the coastal zone including declining water quality, physical disturbance and over-fishing [41]. Within the GBR, recent wide-spread loss of seagrass (from 2008-2011) and record dugong and turtle mortalities were largely attributed to repeated years of above average rainfall and run-off (culminating in extreme weather associated with a category 5 tropical cyclone in February 2011) with associated suspended sediments reducing light available for photosynthetic C-fixation [42,43]. In addition, PSII herbicides have also been detected in runoff entering the GBR lagoon at concentrations above environmental guidelines [2,4] and as such may contribute to losses of coastal seagrasses.

While previous work has identified effects of PSII herbicides on the photophysiology, biochemistry and growth of seagrass (Table 1), there is little reliable quantitative toxicity data for seagrass. Here we applied standard ecotoxicology protocols to quantify the concentrations of four priority PSII herbicides that inhibit photochemistry by 10, 20 and 50% (IC_10_, IC_20_ and IC_50_) over 72 h in two common seagrass species from the GBR lagoon. The time to reach maximum inhibition of photosynthesis by herbicides was also tested using an additional two seagrass species. These data will enable improved assessment of the risks posed by PSII herbicides to tropical seagrass for both regulatory purposes and for comparison with other taxa.

## Materials and Methods

### Herbicides

The four PSII herbicides used in the present study represent three structural groups: (1) the urea herbicides Diuron and Tebuthiuron, (2) the *s-*triazine Atrazine and (3) the trizinone Hexazinone. These herbicides are among the most widely and frequently detected in the GBR lagoon [2–4,44,45].

### Plant collection

Four seagrass species were used in preliminary studies to determine the time taken for PSII herbicides to affect photosynthesis, while more detailed ecotoxicology studies were undertaken with two species as described below. *Halodule* *uninervis* , *Cymodocea* *rotundata* Ascherson (Cymodoceaceae) and *Thalassia* *hemprichii* Ascherson (Hydrocharitaceae) are tropical seagrass species widely distributed throughout the Indo-West Pacific while *Zostera* *muelleri* Irmisch ex Ascherson (Zosteraceae), (syn *Zostera* *capricorni* ) is a tropical to temperate species found in Australia and New Zealand [46]. All four species occur in northeastern Australia and the Great Barrier Reef (GBR). *H. uninervis* *,* *C* *. rotundata* *and* *T* *. hemprichii* were collected from intertidal seagrass meadows (<1 m) from Cockle Bay, Magnetic Island (19°10.88’ S, 146°50.63’ E) while *Z* *. muelleri* was collected from Pelican, Banks, Gladstone, Australia (23°46.005’ S, 151° 18.052E). Seagrasses were collected under permit MTB41, a permit issued for limited impact research in the GBR Marine, Park which was assessed through the Department of Employment, Economic Development and Innovation self-assessable Fisheries Queensland Code MP05 for the removal of marine plants. Plants were transported to the Australian Institute of Marine Science (AIMS) Townsville, Australia in seawater. Pots of all seagrass species in sediment were maintained in outdoor aquaria (1000 l) with flow-through filtered seawater (5 µm) under 70% shading (maximum 350 µmol photons m^-2^ s^-1^), ambient temperature (23-25°C) and salinity at 35-36 PSU.

### Bioassay

Prior to experimentation, plants with 4-9 shoots each were transferred to 500 ml plastic experimental pots of 13.5 x 9.8 cm with a sediment depth of 4.5 cm. These units were placed into 6 l glass aquaria filled with 1 µm filtered seawater, gently aerated and under 273 ± 17 µmol photons m^-2^ s^-1^ (12h light:dark photoperiods, Aqua Illumination LED). This light intensity was chosen as the median daily irradiance at the Magnetic Island collection site [47]. The glass aquaria were placed into water baths and maintained at 25.8 ± 0.3°C (range), equivalent to the annual average temperature in the GBR [48]. Plants were allowed to acclimate for at least one week prior to experimentation. Stock herbicide solutions (5 mg l^-1^ for Diuron, Atrazine and Hexazinone and 50 mg l^-1^ for Tebuthiuron) were prepared in milli-Q (< 0.03% v/v ethanol carrier) and all assays performed in 1 µm filtered seawater. All herbicide standards were >95% pure and were purchased from Sigma-Aldrich.

Initially a series of pilot studies were performed to measure the time it takes for the four PSII herbicides to illicit 90% steady state (maximum) inhibition of effective quantum yield (*ΔF/F*’_*m*_, see below) in *Z* *. muelleri* at single herbicide concentrations. These findings were used to ensure that the exposure duration of later dose-response curves (described below) was sufficient. The nominal herbicide concentrations used were 10 µg l^-1^ Diuron, 50 µg l^-1^ Atrazine, 10 µg l^-1^ Hexazinone and 400 µg l^-1^ Tebuthiuron. We also exposed all four species of seagrass to 10 µg l^-1^ Diuron to examine the consistency of response times between species. Inhibition of *ΔF/F*’_*m*_ by the herbicides compared with carrier controls were conducted at multiple times up to 24 h.

The studies above revealed a rapid response of the seagrass tested to the herbicides so the final series of static seagrass exposure assays with *H* *. uninervis* and *Z* *. muelleri* were performed over 72 h, with 100% water replaced every 24 h. These two species were each exposed to seven elevated concentrations of each herbicide (Table 2) along with seawater and solvent carrier controls. All treatments were conducted in duplicate tanks. After 72 h exposures, *H* *. uninervis* and *Z* *. muelleri* were removed from the experimental containers, washed free of sediment and placed into -20°C for later analysis of growth (see below).

**Table 2:** Measured herbicide concentrations.

**Herbicide**	**Diuron**		**Atrazine**		**Hexazinone**		**Tebuthiuron**	
**Time (h)**	**0**	**72**	**0**	**72**	**0**	**72**	**0**	**72**
Nominal								
0	BRL	BRL	BRL	BRL	BRL	BRL	BRL	BRL
0.12	0.24	0.15	-	-	-	-	-	-
0.37	0.41	0.34	0.37	0.38	0.4	0.39	-	-
1.2	1.09	1.15	1.4	1.32	1.24	1.37	1.49	1.63
3.7	2.91	2.95	3.35	3.50	4.12	4.04	4.34	4.57
12	9.70	9.87	11.5	13.0	15.2	12.9	14.3	8.82
37	28.3	28.6	37.0	35.7	40.2	40.3	43.1	42.0
120	102	87.8	147	122	132	141	140	142
370	-	-	374	365	346	397	394	442
1100	-	-	-	-	-	-	1008	1023

Mean measured herbicide concentrations (µg l^-1^) at the beginning and end of toxicity assays against the nominal concentrations. Seawater and solvent controls were below reporting limit (BRL) of < 0.1 µg l^-1^. Not used (-).

### Chlorophyll fluorescence

Chlorophyll a fluorescence measurements (effective quantum yield, *ΔF/F*’_m_ and maximum quantum yield, *F_v_/F*_*m*_) were taken just prior to the start of exposure and after 24 and 72 h using a pulse amplitude modulated chlorophyll fluorometer (mini-PAM, Walz, Germany). Measurements were obtained by placing a 2 mm fibre-optic probe perpendicular to the surface of the seagrass leaf. Measurements were made on 6-8 leaves per pot with two measurements taken per leaf between 1-2 cm from the top of the sheath. Measurements were made only on green, non-senescent leaves i.e. not showing signs of pigment loss. Initial fluorescence (*F* in illuminated samples and *F*_*0*_ in dark-adapted samples) was determined by applying a weak pulse-modulated red measuring light (650 nm, 0.15 µmol photons m^-2^s^-1^). The light adapted maximum fluorescence (*F*’_*m*_) was quantified by applying a short pulse (800 ms) of saturating actinic light (>3000 µmol photons m^-2^s^-1^). The effective quantum yield in an illuminated plant *(∆F/F*’_*m*_, Eq. 1) provides an estimate of the efficiency of photochemical energy conversion within photosystem II (PSII) under a specific light intensity [49]. The reversible binding of PSII herbicides to the D1 protein in PSII results in an immediate and temporary reduction in *∆F/F*’_*m*_ [22].

ΔF/Fm'=(Fm−F)/Fm' (1)

The maximum quantum yield (*F_v_/F*_*m*_) is equivalent to the proportion of light used for photosynthesis by chlorophyll when all reaction centres are open [49]. A reduction in *F_v_/F*_*m*_, which is measured after a period of dark-adaptation indicates photooxidative damage to PSII (chronic photoinhibition). In the present study, seagrasses were dark adapted for 30 min and *F*_*0*_ and *F*_*m*_ measured (as above) were used to derive maximum quantum yields as per Eq. 2:

Fv/Fm=(Fm−F0)/Fm (2)

The inhibition of *∆F/F*_*m*_ *′* and *F_v_/F*_*m*_ due to the binding of herbicides or damage to the D1 protein in PSII [15] was calculated according to Eq. 3

$$Inhibition(\%)=\left(1-\frac{YieldTreatment}{YieldControl}\right)\times 100$$ (3)

### Growth

Leaf extension rate was used as a proxy for seagrass productivity [50]. A 25-gauge syringe needle was used to puncture the leaves at the top of the leaf sheath of *H* *. uninervis* and *Z* *. muelleri* . The length of growth (mm) which is the distance from the initial mark to scars on new leaves was measured after 3 d under a stereo microscope (16x magnification) using vernier calipers.

### Herbicide analysis

Water samples (2 ml) were taken 1 h after dosing and at 72 h and pipetted into 4 mL amber glass vials then spiked with 10 µL of a surrogate standard, d5-Atrazine (Novachem, Victoria, Australia). The final concentration of the surrogate standard was 5 µgl^-1^ then stored frozen. Thawed herbicide samples were 0.45 µm filtered then analysed by HPLC-MS/MS using an AB/Sciex API5500Q mass spectrometer (AB/Sciex, Concord, Ontario, Canada) equipped with an electrospray (TurboV) interface and coupled to a Shimadzu Prominence HPLC system (Shimadzu Corp., Kyoto, Japan). Column conditions were as follows: Phenomenex Synergi Fusion RP column (Phenomenex, Torrance, CA) 4 µm, 50 x 2.0 mm, 45°C, with a flow rate of 0.4 ml min^-1^. A linear gradient starting at 8% B for 0.5 min was ramped to 100% B in 8 min then held at 100% for 2 min followed by equilibration at 8% B for 2.5 min (A = 1% methanol in HPLC grade water, B = 95% methanol in HPLC grade water, both containing 0.1% acetic acid). The mass spectrometer was operated in the positive ion, multiple reaction-monitoring mode using nitrogen as the collision gas. The limit of detection for this method was typically less than 0.1 µg l^-1^ and the response linear across the concentration range used. Sample sequences were run with a standard calibration at the beginning and end of sequence with additional mid-range standards run every 10 samples. Measured concentrations can be found in Table 2.

### Data Analysis

Photosynthetic yield data were arcsine square root transformed and growth data were square root transformed to meet the assumptions of one-way analysis of variance (ANOVA). Data were then pooled from replicate tanks following nested ANOVA validation with tank as the nested factor. Inhibition of photosynthetic yields was taken relative to carrier control (for all 4 herbicides) as it was found that there was no significant difference between seawater controls and carrier controls.

The time taken for the herbicides to cause a 90% steady state inhibition of *∆F/F*_*m*_′and *F_v_/F*_m_ was calculated by plotting inhibition data against time using a 3-parameter exponential curve (SigmaPlot 11, Systat Software, CA). 90% of maximum inhibition was used as a precise estimate of response time for comparisons between species and herbicides since the maximum (100%) response would need to be estimated from a trailing asymptote. Dose-response curves for the inhibition of *∆F/F*_*m*_′ and *F_v_/F*_m_ data were produced by fitting inhibition data with measured concentrations using a 4 parameter logistic model (SigmaPlot 11). The herbicide inhibition concentrations (IC_xx_) that inhibited *∆F/F*_*m*_ *′* and *F_v_/F*_*m*_ by 10, 20 and 50% (IC_10_, IC_20_ and IC_50,_ respectively) were determined from each curve. Comparisons of IC_x_ values are more valuable than “no observed effect concentrations” (NOEC) or “lowest observed effect concentrations” (LOEC) for estimating reliable biological responses since modelling data to a function across the range of responses minimises large uncertainties inherent in statistically comparing a limited number of discrete response points against a control [51].

## Results

### Time taken to steady state inhibition

**Figure 1:**
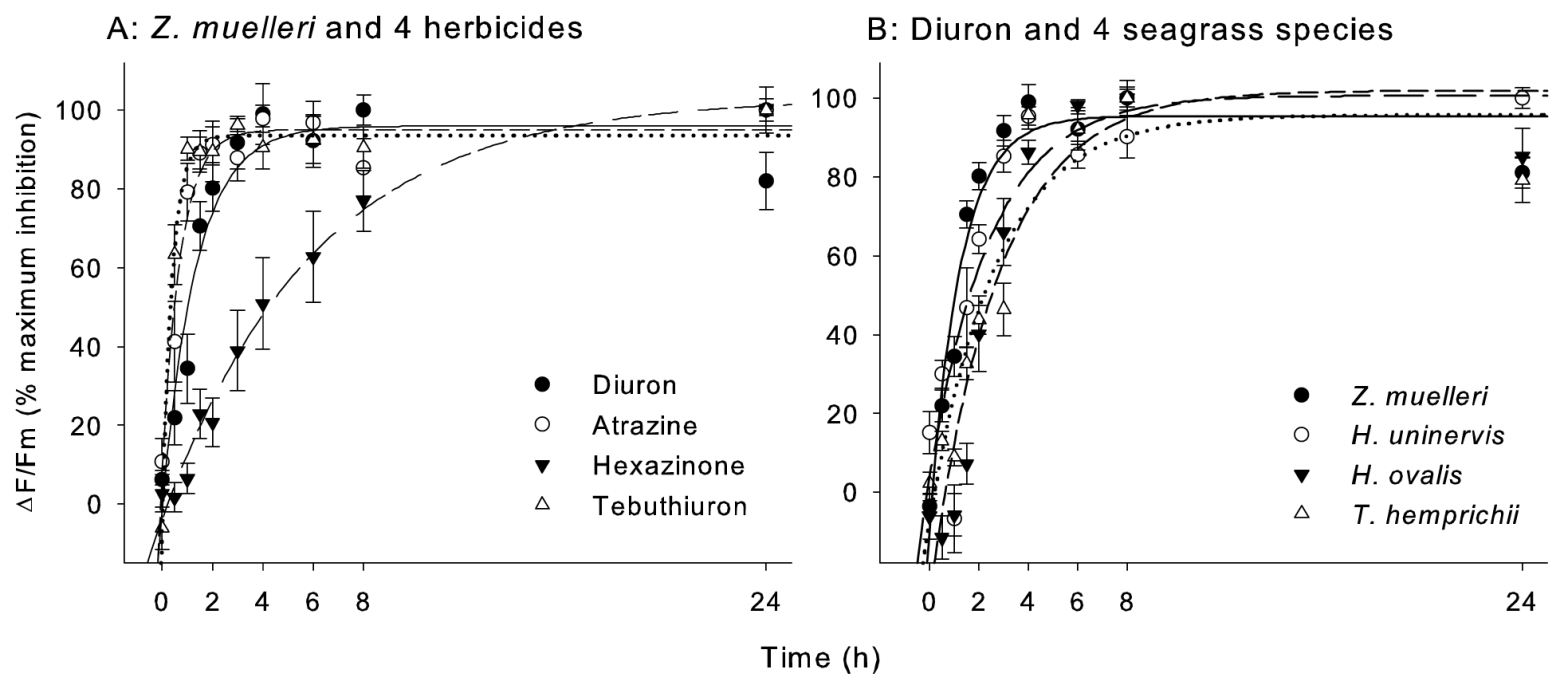
Time taken to steady state inhibition. Inhibition of effective quantum yield (*ΔF/F*’_*m*_) relative to maximum inhibition over time by (A) Diuron (10 µg l^-1^), Atrazine (50 µg l^-1^), Hexazinone (10 µg l^-1^) and Tebuthiuron (400 µg l^-1^) to *Z* *. muelleri* and (B) Diuron (10 µg l^-1^) to four seagrass species. Bars = ± SE, n = 4.

The herbicides Diuron, Atrazine and Tebuthiuron all caused 90% steady state inhibition of effective quantum yield (*ΔF/F*’_*m*_) in *Z* *. muelleri* within 4 hours (Figure 1A, Table 3). Hexazinone acted more slowly on PSII and did not reach 90% of steady state inhibition until almost 13 h (Figure 1A, Table 3). The response of *Z* *. muelleri* to Diuron exposure was more rapid (3.7 hr) than the other three seagrass species tested, with the slowest *T* *. hemprichii* , taking more than twice as long (7.7 hr) to reach 90% steady state inhibition of *ΔF/F*’_*m*_ (Figure 1B, Table 3).

**Table 3:** Time taken to steady state inhibition.

**Species**	*** Z. muelleri ***	*** H. uninervis ***	*** H. ovalis ***	*** T. hemprichii ***
**Herbicide**				
Diuron	3.7	5.5	6.6	7.7
Atrazine	2.0	-	-	-
Hexazinone	12.7	-	-	-
Tebuthiuron	1.5	-	-	-

Time (hours) to 90% of maximum inhibition of effective quantum yield (*ΔF/F*’_*m*_). *Z* *. muelleri* was exposed to four herbicides independently and all four seagrass species were individually exposed to Diuron. Not tested (-).

### Inhibition of effective quantum yield at 72 h

**Figure 2:**
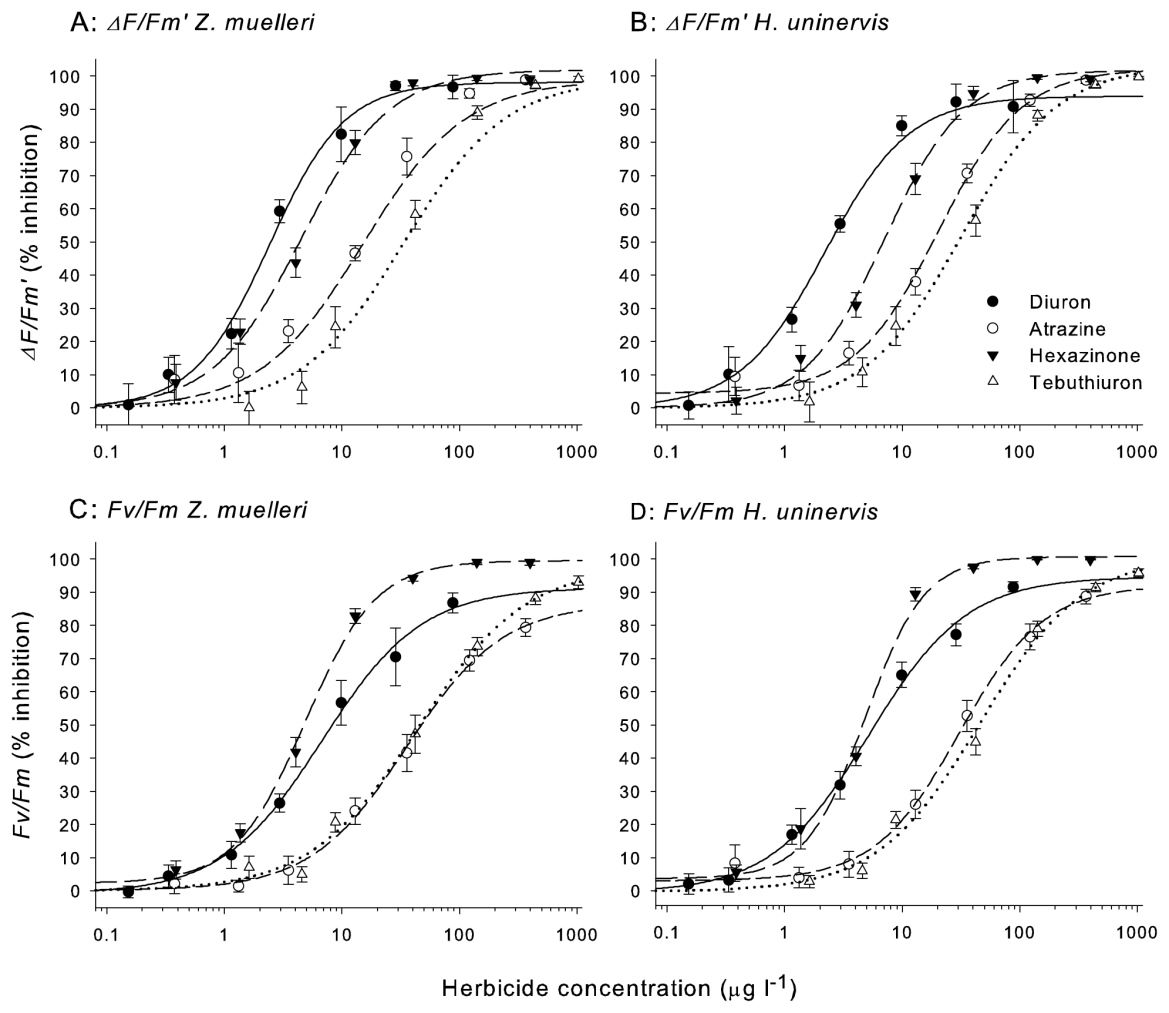
Concentration-response curves for two seagrasses species and four herbicides. Percent inhibition relative to control for effective quantum yield (*ΔF/F*’_*m*_) and maximum potential yields (*F_v_/F_m_*) in *Zostera* *muelleri* and *Halodule* *uninervis* exposed to PSII herbicides over 72 h.

Plots of the inhibition of *ΔF/F*’_*m*_ against concentration for each herbicide-seagrass combination yielded classic sigmoidal dose-response relationships with r^2^ values greater than 0.98 (Figure 2A and 2B). Diuron was consistently the most potent herbicide (lowest IC_50_) to both *Z* *. muelleri* and *H* *. uninervis* followed by Hexazinone, Atrazine and Tebuthiuron (Table 4). Inhibition of *ΔF/F*’_*m*_ was virtually identical for both species exposed to the urea herbicides Diuron and Tebuthiuron. *Z* *. muelleri* on the other hand appeared consistently more sensitive (lower IC_x_) than *H* *. uninervis* to the triazine herbicides Atrazine and Hexazinone (Table 4). No observed effect concentrations (NOEC) for *ΔF/F*’_*m*_ can be found in Table S3.

**Table 4:** Herbicide concentrations that inhibit effective quantum yield in seagrass after 72 h.

	**Diuron**		**Atrazine**		**Hexazinone**		**Tebuthiuron**	
	**IC_50_**	**95% CV**	**IC_50_**	**95% CV**	**IC_50_**	**95% CV**	**IC_50_**	**95% CV**
* Z. muelleri *	2.47	1.96–3.23	13.4	10.5-15.8	4.40	3.50-5.58	29.1	21.7-39.0
* H. uninervis *	2.41	2.04-2.88	18.2	14.1-23.6	6.87	5.54-8.44	29.7	23.8-37.9
	**IC_20_**	**95% CV**	**IC_20_**	**95% CV**	**IC_20_**	**95% CV**	**IC_20_**	**95% CV**
* Z. muelleri *	0.88^99^	0.54-1.23	3.10	2.01-4.13	1.36	0.89-1.88	9.07^90^	6.12-13.0
* H. uninervis *	0.86^99^	0.66-1.10	5.31	3.24-8.01	2.37	1.56-3.33	8.16^90^	5.87-11.4
	**IC_10_**	**95% CV**	**IC_10_**	**95% CV**	**IC_10_**	**95% CV**	**IC_10_**	**95% CV**
* Z. muelleri *	0.49^99^	0.26-0.87	1.17^95^	0.29-1.89	0.67^99^	0.39-1.11	4.79^90^	2.59-7.75
* H. uninervis *	0.47^99^	0.31-0.68	2.11^90^	0.17-4.40	1.27	0.74-2.15	3.87^90^	2.49-6.01

Concentration of herbicides (µg l^-1^) that inhibit effective quantum yield (photosynthetic efficiency *ΔF/F*’_*m*_) by 10%, 20% and 50% (IC_10_, IC_20_ and IC_50_) in *Z* *. muelleri* and *H* *. uninervis* and following 72 h exposures. Results for 24 h exposures can be found in Table S1. Inhibition concentrations (IC_x_) below guideline trigger values for protecting 90%, 95% and 99% of species are indicated by respective superscripts (Table S4 [52]).

### Inhibition of maximum potential quantum yield at 72 h

The inhibition of *F_v_/F*_*m*_ in both seagrass species formed similar sigmoidal relationships with PSII herbicide concentrations (r^2^ > 0.99) (Figure 2C and 2D). However, photosynthetic yields in the dark (*F_v_/F*_*m*_) were not inhibited by Diuron, Atrazine and Tebuthiuron to the same extent (greater IC_50_ values) as those taken in illuminated conditions (*ΔF/F*’_*m*_) (Table 5). Interestingly, the slopes of the *F_v_/F*_*m*_ inhibition curves for Hexazinone were 1.45 ( *Z* *. muelleri* ) and 1.73 ( *H* *. uninervis* ), which were greater than the slopes for the other herbicide-seagrass combinations (0.95-1.21). Consequently, Hexazinone was the most potent inhibitor of *F_v_/F*_*m*_ with IC_50_ values of 4.61 µg l^-1^ ( *Z* *. muelleri* ) and 4.75 µg l^-1^ ( *H* *. uninervis* ), which were similar to their respective light adapted yields (Table 4 and Table 5). No observed effect concentrations (NOEC) for *F_v_/F*_*m*_ can be found in Table S3.

**Table 5:** Herbicide concentrations that inhibit maximum yield in seagrass after 72 h.

	**Diuron**		**Atrazine**		**Hexazinone**		**Tebuthiuron**	
	**IC_50_**	**95% CV**	**IC_50_**	**95% CV**	**IC_50_**	**95% CV**	**IC_50_**	**95% CV**
* Z. muelleri *	8.33	6.58-10.8	47.9	39.8-57.8	4.75	4.06-5.63	46.1	34.2-64.2
* H. uninervis *	5.89	4.69-7.52	33.3	26.1-44.5	4.61	3.57-6.01	44.8	32.7-62.0
	**IC_20_**	**95% CV**	**IC_20_**	**95% CV**	**IC_20_**	**95% CV**	**IC_20_**	**95% CV**
* Z. muelleri *	2.04^90^	1.44-2.75	11.4	8.86-14.1	1.73	1.31-2.23	10.3^90^	6.64-15.5
* H. uninervis *	1.52^95^	1.06-2.02	9.24	5.92-13.4	1.92	1.23-2.81	11.2^90^	6.97-17.5
	**IC_10_**	**95% CV**	**IC_10_**	**95% CV**	**IC_10_**	**95% CV**	**IC_10_**	**95% CV**
* Z. muelleri *	0.95^95^	0.57-1.48	5.14	3.55-7.30	0.92^99^	0.52-1.31	4.39^90^	2.37-7.99
* H. uninervis *	0.70^99^	0.42-1.09	3.98	1.77-7.40	1.03^99^	0.26-1.91	5.03^90^	2.19-9.47

Concentration of herbicides (µg l^-1^) that inhibit maximum potential quantum yield (indicating damage to PSII, *F_v_/F*_*m*_) by 10%, 20% and 50% (IC_10_, IC_20_ and IC_50_) in *Z* *. muelleri* and *H* *. uninervis* and following 72 h exposures. Results for 24 h exposures can be found in Table S2. Inhibition concentrations (IC_x_) below guideline trigger values for protecting 90%, 95% and 99% of species are indicated by respective superscripts (Table S4 [52]).

### Assay duration

Since PAM fluorometry is a non-destructive technique, we were able to measure the responses of both *∆F/F*_*m*_′and *F_v_/F*_m_ to the same herbicides following the first 24 h exposure. There was little difference in inhibition with the IC_x_ values at 24 h (Tables S1 and S2) than those obtained at 72 h (Tables 4 and 5). For example, the mean ratios for IC_50_ (24 h/72 h) for all herbicides and seagrass combinations were 0.96 ± (0.05) SE for *ΔF*/*F*_*m*_′ and 1.00 ± 0.04 (SE) for *F_v_/F*_*m*_.

### Growth

Growth rates (leaf extension) in control treatments ranged between 1.5-3.9 mm day^-1^ for *Z* *. muelleri* and 1.6 and 3.9 mm day^-1^ for *H* *. uninervis* in the four 72 h exposure experiments. No significant differences (results not shown) between the herbicide treatments were observed, most likely due to the short duration of the experiment.

## Discussion

The photosystems of seagrasses *Zostera* *muelleri* and *Halodule* *uninervis* were shown to be at least as sensitive to the PSII herbicides Diuron, Atrazine, Hexazinone and Tebuthiuron as corals and tropical microalgae. The herbicides caused rapid inhibition of effective quantum yield (*∆F/F*_*m*_ *′*), indicating reduced photosynthesis and maximum effective yields (*F_v_/F_m_*) corresponding to chronic damage to PSII. The herbicide concentrations which affected photosynthesis have been exceeded in the GBR lagoon and all of the herbicides inhibited photosynthesis at concentrations lower than the water quality guidelines [52] for 90% species protection.

### Time taken to steady state inhibition of effective quantum yield

The time taken to 90% maximum effect on *∆F/F*_*m*_′in seagrass by Diuron was between 3.7 and 7.7 hours for the four species. Although this inhibition is comparable to the 2 to 4 hours observed for coral symbionts [22], the response of microalgae is faster still, often reaching maximum inhibition within 20 min of exposure [24,53]. In agricultural weeds, PSII herbicides are taken up by the roots and transported through the vascular system to PSII in the leaves. The same mechanism may occur in seagrass, although Schwarzschild et al. [12] demonstrated low sensitivity of the seagrass *Zostera* *marina* exposed to Atrazine through the root-rhizome complex, concluding that these herbicides are more likely to be rapidly transported directly across the semi-permeable cell walls of leaves. Hexazinone was the slowest-acting PSII herbicide tested; taking four-times longer to reach 90% maximum inhibition compared with Diuron and was more than 6-fold slower than Atrazine and Tebuthiuron. A similar result was observed for the gradual effect of Hexazinone (2-3 hours rather than minutes for Diuron) on diatoms and green algae [24,25]. The reason for protracted uptake of Hexazinone may be a lower membrane permeability due to its high water solubility (log K_OW_ = 1.2) relative to the other herbicides (log K_OW_ 1.8-2.6) [54]. The concentrations of each herbicide that inhibited 50% of *∆F/F*_*m*_′ or *F_v_/F*_*m*_ (IC_50_s) were identical following 24 and 72 h exposures (Tables 4, 5, S1 and S2), confirming the consistent binding of herbicides to the D1 protein over this time period and indicating that 24 h is a sufficient duration for this endpoint in future ecotoxicological studies.

### Inhibition of effective quantum yield

The inhibition of effective quantum yield (*∆F/F*_*m*_ *′*

) in the light is an ideal measure of PSII herbicide impacts on seagrass since a reduction in ∆F/F_*m*_′indicates blockage of electron transport in PSII during active photosynthesis (due to binding of the PSII herbicide to the D1 protein), which is proportional to the reduction in photosynthetic energy [18,49]. The decline in ∆F/F_*m*_′ following herbicide exposure therefore provides a direct link to a diminished photosynthetic carbon fixation (energy) and finally productivity and growth [18,55]. Reduction of photosynthetic products including oxygen and ATP in seagrass in the presence of Atrazine [28,56–59] further supports this endpoint as a valid indicator of stress in seagrass. Quantifying the herbicide concentrations which inhibit ∆F/F_*m*_′by 50% (IC_50_) allows comparisons of the potency of PSII herbicides and the sensitivity of different species and taxa to the PSII herbicides; however, IC_50_s for ∆F/F_*m*_′ had not been described for the effects of PSII herbicides on seagrass previously (Table 1). Here we demonstrate that the seagrasses Z. muelleri (IC_50_ = 2.5 µgl^-1^) and H. uninervis (IC_50_ = 2.4 µgl^-1^) were generally more sensitive to the PSII herbicides tested than tropical corals, microalgae, foraminifera, and crustose coralline algae tested in similar experiments (Table 6). While symbionts in the coral Seriatopora hystrix exhibited a similar IC_50_ for Diuron [22], only the green alga Nephroselmis pyriformis had lower IC_50_s for all herbicides [24]. Diuron was the most potent of the PSII herbicides tested (lowest IC_50_) and a comparison of potencies for PSII herbicides can be made for each taxa by comparing the Relative Equivalent Potencies (REP = IC_50 (Diuron)_/IC_50 (PSII herbicide)_) where REP = 1 indicates equal potency as Diuron, while a more potent herbicide will have a REP of >1, and a less potent herbicide REP of <1 [60]. For example, Atrazine had an REP of 0.19 for Z. muelleri, identifying its potency as 19% of the reference herbicide Diuron. Since the PSII herbicides bind to the same receptor, these REP values for seagrass can now be used to combine the contribution of each herbicide in a mixture to a Toxin Equivalent (TEQ) value [24,60,61], enabling comparison of field concentrations with guideline values (Table S4) for assessing risk of herbicides mixtures to seagrass.

**Table 6:** Comparison of IC_50_ and herbicide equivalence values for tropical taxa.

		**Diuron**	**Atrazine**	**Hexazinone**	**Tebuthiuron**	
**Taxa/Species**	**Duration**	**IC_50_ (HEQ)**	**IC_50_ (HEQ)**	**IC_50_ (HEQ)**	**IC_50_ (HEQ)**	**Reference**
**Seagrass**						
* Z. muelleri *	72 h	2.5 (1.0)	13 (0.19)	4.4 (0.57)	29 (0.086)	This study
* H. uninervis *	72 h	2.4 (1.0)	18 (0.13)	6.9 (0.35)	30 (0.080)	This study
**Coral**						
* Acropora millepora *	7 d	2.9 (1.0)	47 (0.062)	14 (0.21)		[19]
* Seriatopora hystrix *	14 h	2.3 (1.0)	45 (0.051)	8.8 (0.26)	175 (0.013)	[21]
* Acropora formosa *	14 h	5.1 (1.0)	37 (0.14)			[22]
* Montipora digitata *	10 h	5.9 (1.0)	88 (0.067)			[22]
* Porites cylindrica *	10 h	4.3 (1.0)	67 (0.064)			[22]
* Seriatopora hystrix *	10 h	2.9				[22]
**Diatom**						
*Navicula sp.*	4 h	2.6 (1.0)	36 (0.072)	5.7 (0.46)	94 (0.028)	[24]
* Cylindrotheca closteriuma *	4 h	4.4 (1.0)	77 (0.057)	6.9 (0.64)	77 (0.057)	[24]
* Phaeodactylum tricornutuma *	4 h	2.7 (1.0)	34 (0.079)	6.6 (0.41)	51 (0.053)	[24]
* Phaeodactylum tricornutuma *	2 h	18 (1.0)	45 (0.40)	22 (0.82)		[25]
**Green alga**						
* Nephroselmis pyriformis *	4 h	2.1 (1.0)	14 (0.15)	2.4 (0.88)	12 (0.18)	[24]
**Foraminifera**						
* Heterostegina depressa *	24 h	11				[26]
**Crustose algae**						
* Neogoneolithon fosliei *	7 d	8.5 (1.0)	180 (0.047)	152 (0.056)		[19]
**All species**						
Mean for all species		5.2 (1.0)	54 (0.12)	23 (0.46)	67 (0.070)	

PSII herbicide concentrations (µg l^-1^) that inhibit effective quantum yield (photosynthetic efficiency *ΔF/F*’_*m*_) by 50% across tropical marine taxa. In brackets are PSII herbicide equivalence values (HEQ) for each herbicide, derived by dividing the IC_50_ of the reference herbicide Diuron by the respective IC_50_ for each herbicide-organism combination. A relative equivalent potency (REP) of 1 indicates equal potency as Diuron while a more potent herbicide will have a REP of >1, and a less potent herbicide REP of <1.

### Inhibition of maximum potential quantum yield

When PSII herbicides bind to the D1 reaction centre in PSII in the presence of moderate-high light, excess energy that cannot be used in photosynthesis is produced. Oxygen radicals are formed as a result and these have the potential to cause photooxidative damage to reaction centres [15,49]. A drop in maximum potential quantum yield (*F_v_/F*_*m*_), which is measured after a period of photosystem “relaxation” in the dark, signifies proportional photoinactivation or damage to PSII. This chronic photoinactivation was observed for all herbicides-seagrass combinations (Table 5) and occurred at slightly greater herbicide concentrations (higher IC_50_s) than the temporary inhibition of *∆F/F*_*m*_′ (Table 4). Two previous studies have reported IC_50_s for *F_v_/F_m_* inhibition in seagrass by Diuron, with identical sensitivity reported over 72 h exposures for *Thalassodendron ciliatum* [10] and a greater sensitivity reported for *Zostera marina* over a 10 day period [35] (Table 6). The impact of herbicide exposure on chronic photoinhibiton (*F_v_/F_m_*) will depend on the duration of exposure, light intensity and the protective mechanisms of the seagrass to deal with oxidative stress and these factors all need to be considered when assessing comparative impacts on seagrass [62]. Hexazinone caused damage to PSII in the seagrass at lower concentrations than the other herbicides as seen by the steeper slopes of the dose response curves, which may signify a positive interaction between Hexazinone with another biochemical or stressor on PSII under the experimental conditions (Fig. 2C and 2D) [63]. Hexazinone also had a strong impact on *F_v_/F*_m_ in coral symbionts [19] and unlike *ΔF/F*’*_m_*, the effects of PSII herbicides in mixtures containing Hexazinone may not be additive for *F_v_/F*_*m*_.

### Whole plant impacts

As described above, exposure to the PSII herbicides is likely to result in starvation over time caused by reductions in electron transport and photosynthetic C-fixation. While the effects of PSII herbicides on photosynthetic efficiency and damage to photosystem II (as measured using PAM fluorometry) are the most sensitive measures of stress on seagrass, exposure to these herbicides has also been shown to cause whole plant effects (Table 1). Reductions in growth of *Z* *. marina* were observed at Diuron concentrations as low as 5 µg l^-1^ over 10 days [35] and Atrazine concentrations as low as 10 µg l^-1^ over 4 weeks [33]. We did not observe inhibition of seagrass growth following 72 h exposures for any of the herbicides tested but this is not surprising as the duration of exposure was likely too short to deplete the plant’s energy reserves. These reserves are carbohydrates (principally starch, and some soluble sugars) in the rhizomes, which can sustain growth in *H* *. uninervis* and *Z* *. muelleri* for more than a month even under extremely reduced rates of C-fixation (such as light stress) [64,65]. Furthermore, although strong reductions in photosynthetic efficiency were measured in the present study, the seagrass would still be able to fix some carbon in most treatments.

### Multiple impacts

Results from this study are conservative, as the seagrass in our experiments were exposed to a moderate light intensity of 280 µE to reflect the median irradiance at the Magnetic Island collection site [47] and were not thermally stressed. Future growth and survival studies should take into account the likelihood that seagrasses are exposed to PSII herbicides under a range of environmental extremes associated with riverine run-off during summer monsoonal conditions. These added or cumulative impacts could increase the effect of PSII herbicide exposure at the whole plant level. For example, low light conditions tend to occur in flood plumes that simultaneously deliver herbicides and light-reducing suspended solids into seagrass meadows, and the combined effect of low light and PSII herbicide exposure would likely lead to more extreme impacts on plant C-fixation. However, seagrass can also grow in intertidal habitat (which is particularly common in the GBR) where they are also periodically exposed to extremely high (full sun) light levels, which can add oxidative stress. For example, Delistraty and Hershner [59] reported growth inhibition in response to 100 µg l^-1^ Atrazine under high light conditions of 500-1000 µE m^-2^s^-1^ and mortality (mostly likely due to oxidative stress) was observed after as little as 7 days.

### Environmental relevance

The current Australian guidelines for ecosystem protection from the PSII herbicides are listed in Table S4 and are not always protective of the effects of these herbicides on seagrass. For example, the effective quantum yield (*∆F/F*_*m*_ *′*) was inhibited by more than 20% in both seagrass species for Diuron and Tebuthiuron and by 10% for Atrazine and Hexazinone exposures at concentrations below the GBRMPA 2010 [52] guidelines for 90% species protection (Table 4). Diuron and Hexazinone also inhibited ∆F/F_*m*_′ in Z. muelleri at concentrations below the 99% species protection guideline which is currently applied to this World Heritage Area [52]. Damage to PSII in seagrass (F_*v*_/F_*m*_) was also apparent for concentrations of Diuron, Hexazinone and Tebuthiuron below these guidelines (Table 5). While inhibition of photosynthetic processes in seagrass for short durations may not represent a catastrophic habitat impact, they do signify a direct and legitimate physiological impact that is likely to add to other simultaneous stresses faced by this foundation taxon. Even ignoring additional stressors, the combined concentrations of PSII herbicides detected in estuarine and marine waters of the GBR lagoon during the wet season have exceeded both the regulatory guidelines [2,4,66] and concentrations that inhibit photosynthetic efficiency in seagrass (this study). Furthermore, herbicides are found in estuarine sediment interstitial waters at concentrations exceeding the water column, even in the dry season [67], and therefore in situ uptake through the root-rhizome complex could contribute to chronic impacts. While all of the PSII herbicides in the present study can contribute to seagrass toxicity, the relative frequency and detection at toxic concentrations, combined with its high potency (Table 6) renders Diuron the PSII herbicide most likely to impact upon estuarine and coastal waters of the GBR.

The greatest wide-spread threat to seagrass populations on the northern coast of Australia, including the GBR, is light limitation due to high levels of suspended solids, resulting from flood plumes and resuspension [43,68]. There is a strong likelihood that the impacts of light limitation from flood plumes and reduced photosynthesis from PSII herbicides exported in the same waters would combine to affect seagrass productivity. Other stressors such as increased sea surface temperatures have been shown to combine with herbicides to increase the effects on coral symbionts [19], but this remains untested for seagrass. Further research is needed to quantitatively link the chronic effects of PSII herbicides on photophysiology, growth and mortality under low light and salinity and high temperature scenarios experienced during monsoonal floods. Given that PSII herbicides can affect seagrass at environmental concentrations, and that seagrasses grow in coastal and estuarine habitats with a demonstrated risk of exposure to herbicides [2,4], we suggest that revision of environmental guidelines and continued efforts to reduce PSII herbicide concentrations in floodwaters may both help protect seagrass meadows of the GBR from further decline.

## Supporting Information

Table S1 **Herbicide concentrations that inhibit effective quantum yield in seagrass after 24 h.** Concentration of herbicides that inhibit effective quantum yield (photosynthetic efficiency *ΔF/F*’_*m*_) by 10%, 20% and 50% (IC_10_, IC_20_ and IC_50_) in *H* *. uninervis* and *Z* *. muelleri* following 24 h exposures.
(DOCX)

Table S2 **Herbicide concentrations that inhibit maximum yield in seagrass after 24 h.** Concentration of herbicides that inhibit maximum potential quantum yield (indicating damage to PSII, *F_v_/F*_*m*_) by 10%, 20% and 50% (IC_10_, IC_20_ and IC_50_) in *H* *. uninervis* and *Z* *. muelleri* following 24 h exposures.
(DOCX)

Table S3 **No observed effect concentrations.** No observed effect concentrations (NOEC, µg l^-1^) values from nested one-way ANOVA (p < 0.05).
(DOCX)

Table S4 **Australian guidelines trigger values for ecological protection.**
(DOCX)

## References

[1] Haynes D, Muller J, Carter S (2000) Pesticide and herbicide residues in sediments and seagrasses from the Great Barrier Reef World Heritage Area and Queensland Coast. Mar Pollut Bull 41: 279-287. doi:10.1016/S0025-326X(00)00097-7.

[2] Lewis SE, Brodie JE, Bainbridge ZT, Rohde KW, Davis AM et al. (2009) Herbicides: A new threat to the Great Barrier Reef. Environ Pollut 157: 2470-2484. doi:10.1016/j.envpol.2009.03.006. PubMed: 19349104. 19349104 10.1016/j.envpol.2009.03.006

[3] Kennedy K, Schroeder T, Shaw M, Haynes D, Lewis S et al. (2012) Long term monitoring of photosystem II herbicides – Correlation with remotely sensed freshwater extent to monitor changes in the quality of water entering the Great Barrier Reef, Australia. Mar Pollut Bull 65: 292-305. doi:10.1016/j.marpolbul.2011.10.029. PubMed: 22154275. 22154275 10.1016/j.marpolbul.2011.10.029

[4] Lewis SE, Schaffelke B, Shaw M, Bainbridge ZT, Rohde KW et al. (2012) Assessing the additive risks of PSII herbicide exposure to the Great Barrier Reef. Mar Pollut Bull 65: 280-291. doi:10.1016/j.marpolbul.2011.11.009. PubMed: 22172236. 22172236 10.1016/j.marpolbul.2011.11.009

[5] Smith R, Middlebrook R, Turner R, Huggins R, Vardy S et al. (2012) Large-scale pesticide monitoring across Great Barrier Reef catchments – Paddock to Reef Integrated Monitoring, Modelling and Reporting Program. Mar Pollut Bull 65: 117-127. doi:10.1016/j.marpolbul.2011.08.010. PubMed: 21920563. 21920563 10.1016/j.marpolbul.2011.08.010

[6] Shaw M, Müller JF (2005) Preliminary evaluation of the occurrence of herbicides and PAHs in the Wet Tropics region of the Great Barrier Reef, Australia, using passive samplers. Mar Pollut Bull 51: 876–881. doi:10.1016/j.marpolbul.2005.04.015. PubMed: 15919098. 15919098 10.1016/j.marpolbul.2005.04.015

[7] Lewis SE, Smith R, Brodie JE, Bainbridge ZT, Davis AM et al. (2011) Using monitoring data to model herbicides exported to the Great Barrier Reef, Australia. In Chan F Marinova D Anderssen RS MODSIM 2011, 19th International Congress on Modelling and Simulation. Modelling and Simulation Society of Australia and New Zealand, 12 2011, : 2051-2056. Available: www.mssanz.org.au/modsim2011/E5/lewis.pdf. Accessed 26 June 2013.

[8] Waters DK, Carroll C, Ellis R, Hateley L, McCloskey J et al. (2013) Modelling reductions of pollutant loads due to improved management practices in the Great Barrier Reef Catchments - Whole of GBR, Volume 1 Department of Natural Resources and Mines. Technical Report (ISBN: 978-1-7423-0999).

[9] Kroon FJ, Kuhnert PM, Henderson BL, Wilkinson SN, Kinsey-Henderson A et al. (2012) River loads of suspended solids, nitrogen, phosphorus and herbicides delivered to the Great Barrier Reef lagoon. Mar Pollut Bull 65: 167-181. doi:10.1016/j.marpolbul.2011.10.018. PubMed: 22154273. 22154273 10.1016/j.marpolbul.2011.10.018

[10] Wahedally S, Mamboya F, Lyimo T, Bhikajee M, Björk M (2012) Short-term effects of three herbicides on the maximum quantum yield and electron transport rate of tropical seagrass *Thalassodendron* *ciliatum* . Tanzania J. Natural App. Sci. 3: 458-466.

[11] Balakrishnan S, Takeda K, Sakugawa H (2012) Occurrence of Diuron and Irgarol in seawater, sediments and planktons of Seto Inland Sea, Japan. Geochem J 46: 169-177.

[12] Schwarzschild AC, MacIntyre WG, Moore KA, Laurence Libelo E (1994) *Zostera* *marina* L. growth response to atrazine in root-rhizome and whole plant exposure experiments. J Exp Mar Biol Ecol 183: 77-89. doi:10.1016/0022-0981(94)90158-9.

[13] Barrett K, Jaward FM (2012) A review of endosulfan, dichlorvos, diazinon, and diuron – pesticides used in Jamaica. Int J Environ Health Res 22: 481-499. doi:10.1080/09603123.2012.667794. PubMed: 22720746. 22720746 10.1080/09603123.2012.667794

[14] Oettmeier W (1992) Herbicides of photosystem II. In: Barber J The Photosystems: Structure, Function and Molecular Biology. Amsterdam: Elsevier pp. 349-408.

[15] Osmond CB, Anderson JM, Ball MC, Egerton JJG (1999) Compromising efficiency: the molecular ecology of light resource utilisation in terrestrial plants. In: Scholes C Baker M Advances in physiological plant ecology. Oxford: Blackwell Publishing House pp. 1-24.

[16] Schreiber U, Bilger W, Neubauer C (1994) Chlorophyll fluorescence as a non-intrusive indicator for rapid assessment of in vivo photosynthesis. In: Schulze ED Caldwell MM Ecophysiology of Photosynthesis. Berlin: Springer-Verlag pp. 49-70.

[17] Jones RJ (2005) The ecotoxicological effects of Photosystem II herbicides on corals. Mar Pollut Bull 51: 495-506. doi:10.1016/j.marpolbul.2005.06.027. PubMed: 16054161. 16054161 10.1016/j.marpolbul.2005.06.027

[18] Ralph PJ, Smith RA, Macinnis-Ng CMO, Seery CR (2007) Use of fluorescence-based ecotoxicological bioassays in monitoring toxicants and pollution in aquatic systems: Review. Toxicol. Environ. Chem 89: 589-607.

[19] Negri AP, Flores F, Röthig T, Uthicke S (2011) Herbicides increase the vulnerability of corals to rising sea surface temperature. Limnol. Oceanog. 56: 471-485. doi:10.4319/lo.2011.56.2.0471.

[20] Shaw CM, Lam PKS, Mueller JF (2008) Photosystem II herbicide pollution in Hong Kong and its potential photosynthetic effects on corals. Mar Pollut Bull 57: 473-478. doi:10.1016/j.marpolbul.2008.04.002. PubMed: 18486951. 18486951 10.1016/j.marpolbul.2008.04.002

[21] Jones RJ, Muller J, Haynes D, Schreiber U (2003) Effects of herbicides diuron and atrazine on corals of the Great Barrier Reef, Australia. Mar Ecol Prog Ser 251: 153-167. doi:10.3354/meps251153.

[22] Jones RJ, Kerswell AP (2003) Phytotoxicity of photosystem II (PSII) herbicides to coral. Mar Ecol Prog Ser 261: 149-159. doi:10.3354/meps261149.

[23] Bengtson Nash SM, McMahon K, Eaglesham G, Müller JF (2005) Application of a novel phytotoxicity assay for the detection of herbicides in Hervey Bay and the Great Sandy Straits. Mar Pollut Bull 51: 351-360. doi:10.1016/j.marpolbul.2004.10.017. PubMed: 15757734. 15757734 10.1016/j.marpolbul.2004.10.017

[24] Magnusson M, Heimann K, Quayle P, Negri AP (2010) Additive toxicity of herbicide mixtures and comparative sensitivity of tropical benthic microalgae. Mar Pollut Bull 60: 1978-1987. doi:10.1016/j.marpolbul.2010.07.031. PubMed: 20800855. 20800855 10.1016/j.marpolbul.2010.07.031

[25] Muller R, Schreiber U, Escher BI, Quayle P, Bengtson Nash SM, et al. (2008) Rapid exposure assessment of PSII herbicides in surface water using a novel chlorophyll a fluorescence imaging assay. Sci Total Environ 401: 51-59. doi:10.1016/j.scitotenv.2008.02.062. PubMed: 18501956. 18501956 10.1016/j.scitotenv.2008.02.062

[26] van Dam JW, Negri AP, Mueller JF, Uthicke S (2012) Symbiont-specific responses in foraminifera to the herbicide diuron. Mar Pollut Bull 65: 373-383. doi:10.1016/j.marpolbul.2011.08.008. PubMed: 21917276. 21917276 10.1016/j.marpolbul.2011.08.008

[27] Bayley S, Stotts V, Springer P, Steenis J (1978) Changes in submerged aquatic macrophyte populations at the head of Chesapeake Bay, 1958–1975. Estuaries 1: 171-182. doi:10.2307/1351459.

[28] Correll DL, Wu TL (1982) Atrazine toxicity to submersed vascular plants in simulated estuarine microcosms. Aquat Bot 14: 151-158. doi:10.1016/0304-3770(82)90094-8.

[29] Den Hartog C, Polderman PJG (1975) Changes in the seagrass populations of the Dutch Waddenzee. Aquat Bot 1: 141-147. doi:10.1016/0304-3770(75)90019-4.

[30] McMahon K, Bengtson Nash S, Eaglesham G, Müller JF, Duke NC et al. (2005) Herbicide contamination and the potential impact to seagrass meadows in Hervey Bay, Queensland, Australia. Mar Pollut Bull 51: 325-334. doi:10.1016/j.marpolbul.2004.10.045. PubMed: 15757731. 15757731 10.1016/j.marpolbul.2004.10.045

[31] Ralph PJ (2000) Herbicide toxicity of *Halophila* *ovalis* assessed by chlorophyll a fluorescence. Aquat Bot 66: 141-152. doi:10.1016/S0304-3770(99)00024-8.

[32] Haynes D, Ralph P, Prange J, Dennison B (2000) The impact of the herbicide diuron on photosynthesis in three species of tropical seagrass. Mar Pollut Bull 41: 288-293. doi:10.1016/S0025-326X(00)00127-2.

[33] Gao Y, Fang J, Zhang J, Ren L, Mao Y et al. (2011) The impact of the herbicide atrazine on growth and photosynthesis of seagrass, *Zostera* *marina* ( L.), seedlings. Mar. Pollut. Bull. 62: 1628-1631.. 21724206 10.1016/j.marpolbul.2011.06.014

[34] Devault AD, Pascaline H (2013) Herbicide impact on seagrass communities, in Herbicides - Current Research and Case Studies in Use, Chapter 14, Inetch Open. Science, 340: 353-375. Available: http://www.intechopen.com/books/herbicides-current-research-and-case-studies-in-use/herbicide-impact-on-seagrass-communities. Accessed 26 6 2013.

[35] Chesworth JC, Donkin ME, Brown MT (2004) The interactive effects of the antifouling herbicides Irgarol 1051 and Diuron on the seagrass *Zostera* *marina* ( L.). Aquat. Toxicol. 66: 293-305. 15129771 10.1016/j.aquatox.2003.10.002

[36] Costanza R, d’Arge R, de Groot R, Farber S, Grasso M et al. (1997) The value of the worlds ecosystem services and natural capital. Nature 387: 253-260. doi:10.1038/387253a0.

[37] Marsh H, O’Shea TJ, Reynolds JE III (2012) Ecology and conservation of the sirenia. Cambridge: Cambridge University Press. 521pp.

[38] Cullen-Unsworth L, Unsworth R (2013) Seagrass meadows, ecosystem services, and sustainability. Environ: Sci. Policy. Sustain Dev 55: 14-28.

[39] Heck KL, Carruthers TJB, Duarte CM, Hughes AR, Kendrick G et al. (2008) Trophic transfers from seagrass meadows subsidize diverse marine and terrestrial consumers. Ecosystems 11: 1198-1210. doi:10.1007/s10021-008-9155-y.

[40] Waycott M, Duarte CM, Carruthers TJB, Orth RJ, Dennison WC et al. (2009) Accelerating loss of seagrasses across the globe threatens coastal ecosystems. Proc Natl Acad Sci USA 106: 12377-12381. doi:10.1073/pnas.0905620106. PubMed: 19587236. 19587236 10.1073/pnas.0905620106 PMC2707273

[41] Orth RJ, Carruthers TJB, Dennison WC, Duarte CM, Fourqurean JW et al. (2006) A global crisis for seagrass ecosystems. BioScience 56: 987-996. doi:10.1641/0006-3568(2006)56[987:AGCFSE]2.0.CO;2.

[42] Meager JJ, Limpus CJ (2012) Marine wildlife stranding and mortality database annual report 2011. I. Dugong. Department of the Environment and Heritage Protection, QLD, Australia . pp. 1-30. Available: http://www.ehp.qld.gov.au/wildlife/pdf/dugong-report-2011.pdf . Accessed 26 June 2013

[43] Collier CJ, Waycott M, McKenzie LJ (2012) Light thresholds derived from seagrass loss in the coastal zone of the northern Great Barrier Reef, Australia. Ecol Indic 23: 211-219. doi:10.1016/j.ecolind.2012.04.005.

[44] Shaw M, Furnas MJ, Fabricius K, Haynes D, Carter S et al. (2010) Monitoring pesticides in the Great Barrier Reef. Mar Pollut Bull 60: 113-122. doi:10.1016/j.marpolbul.2009.08.026. PubMed: 19818971. 19818971 10.1016/j.marpolbul.2009.08.026

[45] Packett R, Dougall C, Rohde K, Noble R (2009) Agricultural lands are hot-spots for annual runoff polluting the southern Great Barrier Reef lagoon. Mar Pollut Bull 58: 976-986. doi:10.1016/j.marpolbul.2009.02.017. PubMed: 19303607. 19303607 10.1016/j.marpolbul.2009.02.017

[46] Waycott M, McMahon KM, Mellors JE, Calladine A, Kleine D (2004) A guide to tropical Seagrasses of the Indo-West Pacific. Townsville: James Cook University p. 72.

[47] Collier CJ, Uthicke S, Waycott M (2011) Thermal tolerance of two seagrass species at contrasting light levels Implications for future distribution in the Great Barrier Reef. Limnol Oceanogr 56: 2200-2210. doi:10.4319/lo.2011.56.6.2200.

[48] Lough J (2007) Climate and climate change on the Great Barrier Reef. In: Johnson J Marshall P Climate change and the Great Barrier Reef. Townsville: Great Barrier Reef Marine Park Authority pp. 15-50.

[49] Genty B, Briantais JM, Baker NR (1989) The relationship between the quantum yield of photosynthetic electron transport and quenching of chlorophyll fluorescence. Biochim Biophys Acta 990: 87-92. doi:10.1016/S0304-4165(89)80016-9.

[50] Short FT, Duarte CM (2001) Methods for the measurement of seagrass growth and production. Global seagrass research methods. 155-182. Available: http://www.chesapeake.org/SAV/literature/01Short.pdf. Accessed 26 June 2013.

[51] Warne M St J; van Dam R (2008) NOEC and LOEC data should no longer be generated or used. Aust. J. Ecotoxicol. 14: 1-5.

[52] GBRMPA (2010) Water quality guidelines for the Great Barrier Reef Marine, Park (Revised). Great Barrier Reef Marine Park Authority, Townsville. Available: http://www.gbrmpa.gov.au/corp_site/key_issues/water_quality/water_quality_guidelines. Accessed 26 June 2013.

[53] Schreiber U, Quayle P, Schmidt S, Escher BI, Mueller JF (2007) Methodology and evaluation of a highly sensitive algae toxicity test based on multiwell chlorophyll fluorescence imaging. Biosens Bioelectron 22: 2554–2563. doi:10.1016/j.bios.2006.10.018. PubMed: 17118646. 17118646 10.1016/j.bios.2006.10.018

[54] Tomlin CDS (2000) The Pesticide Manual: A World Compendium (12th Edition). Farnham, Surrey, UK: British Crop Protection Council. 1250pp.

[55] Scarlett A, Donkin P, Fileman TW, Evans SV, Donkin ME (1999) Risk posed by the antifouling agent Irgarol 1051 to the seagrass, *Zostera* *marina* . Aquat Toxicol 45: 159-170. doi:10.1016/S0166-445X(98)00098-8.

[56] Walsh GE, Hansen DL, Lawrence DA (1982) A flow-through system for exposure of seagrass to pollutants. Mar Environ Res 7: 1-11. doi:10.1016/0141-1136(82)90047-2.

[57] Jones TW, Winchell L (1984) Uptake and Photosynthetic Inhibition by Atrazine and its Degradation Products on Four Species of Submerged Vascular Plants1. J Environ Qual 13: 243-247. doi:10.2134/jeq1984.132243x.

[58] Johnson JR, Bird KT (1995) The effects of the herbicide Atrazine on *Ruppia* *maritima* L. growing in autotrophic versus heterotrophic cultures. Bot Marina 38: 307-312.

[59] Delistraty DA, Hershner C (1984) Effects of the herbicide atrazine on adenine nucleotide levels in *Zostera* marina L. (eelgrass). Aquat. Bot. 18: 353-369.

[60] Escher BI, Bramaz N, Mueller JF, Quayle P, Rutishauser S et al. (2008) Toxic equivalent concentrations (TEQs) for baseline toxicity and specific modes of action as a tool to improve interpretation of ecotoxicity testing of environmental samples. J Environ Monit 10: 612-621. doi:10.1039/b800949j. PubMed: 18449398. 18449398 10.1039/b800949j

[61] Bengtson Nash SM, Schreiber U, Ralph PJ, Müller JF (2005) The combined SPE:ToxY-PAM phytotoxicity assay; application and appraisal of a novel biomonitoring tool for the aquatic environment. Biosens Bioelectron 20: 1443-1451. doi:10.1016/j.bios.2004.09.019. PubMed: 15590302. 15590302 10.1016/j.bios.2004.09.019

[62] Jones RJ (2005) Testing the "photoinhibition" model of coral bleaching using chemical inhibitors. Mar Ecol Prog Ser 284: 133-145.

[63] Weiss JN (1997) The Hill equation revisited: uses and misuses. FASEB J 11: 835-841. PubMed: 9285481. 9285481

[64] Collier CJ, Waycott M, Ospina AG (2012) Responses of four Indo-West Pacific seagrass species to shading. Mar Pollut Bull 65: 342-354. doi:10.1016/j.marpolbul.2011.06.017. PubMed: 21741666. 21741666 10.1016/j.marpolbul.2011.06.017

[65] Longstaff BJ, Dennison WC (1999) Seagrass survival during pulsed turbidity events: the effects of light deprivation on the seagrasses *Halodule* *pinifolia* and *Halophila* *ovalis* . Aquat Bot 65: 105-121. doi:10.1016/S0304-3770(99)00035-2.

[66] Davis AM, Lewis SE, Bainbridge ZT, Glendenning L, Turner RDR et al. (2012) Dynamics of herbicide transport and partitioning under event flow conditions in the lower Burdekin region, Australia. Mar Pollut Bull 65: 182-193. doi:10.1016/j.marpolbul.2011.08.025. PubMed: 21937063. 21937063 10.1016/j.marpolbul.2011.08.025

[67] Magnusson M, Heimann K, Ridd M, Negri AP (. (2013)) Pesticide contamination and phytotoxicity of sediment interstitial water to tropical benthic microalgae. Water Res, 47: 5211–21. doi:10.1016/j.watres.2013.06.003. PubMed: 23870432. 23870432 10.1016/j.watres.2013.06.003

[68] Fabricius KE, De’ath G, Humphrey C, Zagorskis I, Schaffelke B (2013) Intra-annual variation in turbidity in response to terrestrial runoff on near-shore coral reefs of the Great Barrier Reef. Estuar. Coast. Shelf Sci. 116: 57-65. doi:10.1016/j.ecss.2012.03.010.

[69] Macinnis-Ng CMO, Ralph PJ (2004) In situ impact of multiple pulses of metal and herbicide on the seagrass, *Zostera* *capricorni* . Aquat Toxicol 67: 227-237. doi:10.1016/j.aquatox.2004.01.012. PubMed: 15063073. 15063073 10.1016/j.aquatox.2004.01.012

