# Supplementary material for: Phytotoxicity of Four Photosystem II Herbicides to Tropical Seagrasses
Source: PLoS One. 2013 Sep 30;8(9):e75798. doi: 10.1371/journal.pone.0075798 (PMC3786934; doi:10.1371/journal.pone.0075798)
Supplement: Table S1 — Herbicide concentrations that inhibit effective quantum yield in seagrass after 24 h. Concentration of herbicides that inhibit effective quantum yield (photosynthetic efficiency ΔF/F’m) by 10%, 20% and 50% (IC10, IC20 and IC50) in H . uninervis and Z . muelleri following 24 h exposures. (DOCX) [file pone.0075798.s001.docx]

**Table S1.** **Herbicide concentrations that inhibit effective quantum yield in seagrass after 24 h.** Concentration of herbicides that inhibit effective quantum yield (photosynthetic efficiency *ΔF/F’_m_*) by 10%, 20% and 50% (IC_10_, IC_20_ and IC_50_) in *H. uninervis* and *Z. muelleri* following 24 h exposures.

|  | **Diuron** |  | **Atrazine** |  | **Hexazi.** |  | **Tebuthi.** |  |
| --- | --- | --- | --- | --- | --- | --- | --- | --- |
|  | **IC_50_** | **95% CV** | **IC_50_** | **95% CV** | **IC_50_** | **95% CV** | **IC_50_** | **95% CV** |
| *Z. muelleri* | 2.42 | 2.10-2.82 | 16.5 | 9.18-32.4 | 4.39 | 3.28-5.80 | 23.0 | 20.7-25.7 |
| *H. uninervis* | 2.35 | 2.07-2.66 | 16.6 | 13.9-20.3 | 6.37 | 5.54-7.33 | 25.1 | 21.3-29.6 |
|  | **IC_20_** | **95% CV** | **IC_20_** | **95% CV** | **IC_20_** | **95% CV** | **IC_20_** | **95% CV** |
| *Z. muelleri* | 1.15 | 0.91-1.39 | 7.21 | 3.18-13.25 | 1.45 | 0.88-2.20 | 8.82 | 7.44-10.3 |
| *H. uninervis* | 0.75 | 0.62-0.88 | 6.52 | 4.77-8.85 | 2.40 | 1.91-2.94 | 8.18 | 6.26-10.3 |
|  | **IC_10_** | **95% CV** | **IC_10_** | **95% CV** | **IC_10_** | **95% CV** | **IC_10_** | **95% CV** |
| *Z. muelleri* | 0.76 | 0.57-1.01 | 4.85 | 1.45-10.22 | 0-1.27 | 0.61 | 5.00 | 3.92-6.31 |
| *H. uninervis* | 0.38 | 0.29-0.49 | 3.36 | 1.71-5.44 | 1.30 | 1.92-1.75 | 4.11 | 2.74-5.85 |
