# Supplementary material for: Phytotoxicity of Four Photosystem II Herbicides to Tropical Seagrasses
Source: PLoS One. 2013 Sep 30;8(9):e75798. doi: 10.1371/journal.pone.0075798 (PMC3786934; doi:10.1371/journal.pone.0075798)
Supplement: Table S2 — Herbicide concentrations that inhibit maximum yield in seagrass after 24 h. Concentration of herbicides that inhibit maximum potential quantum yield (indicating damage to PSII, Fv/F m) by 10%, 20% and 50% (IC10, IC20 and IC50) in H . uninervis and Z . muelleri following 24 h exposures. (DOCX) [file pone.0075798.s002.docx]

**Table S2.** **Herbicide concentrations that inhibit maximum yield in seagrass after 24 h.** Concentration of herbicides that inhibit maximum potential quantum yield (indicating damage to PSII, *F_v_/F_m_*) by 10%, 20% and 50% (IC_10_, IC_20_ and IC_50_) in *H. uninervis* and *Z. muelleri* following 24 h exposures.

|  | **Diuron** |  | **Atrazine** |  | **Hexazi.** |  | **Tebuthi.** |  |
| --- | --- | --- | --- | --- | --- | --- | --- | --- |
|  | **IC_50_** | **95% CV** | **IC_50_** | **95% CV** | **IC_50_** | **95% CV** | **IC_50_** | **95% CV** |
| *Z. muelleri* | 9.86 | 9.04-10.9 | 49.9 | 41.0-61.0 | 4.51 | 4.28-4.73 | 44.1 | 38.5-50.7 |
| *H. uninervis* | 6.03 | 4.63-8.24 | 27.1 | 22.7-33.2 | 4.45 | 3.99-4.96 | 49.1 | 39.8-62.5 |
|  | **IC_20_** | **95% CV** | **IC_20_** | **95% CV** | **IC_20_** | **95% CV** | **IC_20_** | **95% CV** |
| *Z. muelleri* | 2.05 | 1.80-2.34 | 8.04 | 6.47-9.93 | 1.62 | 1.48-1.76 | 10.0 | 8.1-12.1 |
| *H. uninervis* | 2.09 | 1.45-2.85 | 7.39 | 5.54- 9.55 | 2.09 | 1.76-2.42 | 13.4 | 9.6-18.0 |
|  | **IC_10_** | **95% CV** | **IC_10_** | **95% CV** | **IC_10_** | **95% CV** | **IC_10_** | **95% CV** |
| *Z. muelleri* | 0.83 | 0.67-0.99 | 3.10 | 2.22-4.29 | 0.88 | 0.76-0.98 | 4.02 | 2.76-5.28 |
| *H. uninervis* | 1.17 | 0.72-1.84 | 3.55 | 2.34-5.16 | 1.33 | 1.05-1.65 | 6.22 | 3.74-9.79 |
