# Supplementary material for: Phytotoxicity of Four Photosystem II Herbicides to Tropical Seagrasses
Source: PLoS One. 2013 Sep 30;8(9):e75798. doi: 10.1371/journal.pone.0075798 (PMC3786934; doi:10.1371/journal.pone.0075798)
Supplement: Table S3 — No observed effect concentrations. No observed effect concentrations (NOEC, µg l-1) values from nested one-way ANOVA (p < 0.05). (DOCX) [file pone.0075798.s003.docx]

**Table S3. No observed effect concentrations.**

No observed effect concentrations (NOEC, µg l^-1^) values from nested one-way ANOVA (p < 0.05).

| **Species** | **Herbicide** | ***ΔF/F’_m_*** | ***F_v_/F_m_*** |
| --- | --- | --- | --- |
| *Z. muelleri* | Diuron | 0.34 | 0.34 |
|  | Atrazine | 3.5 | 3.5 |
|  | Hexazinone | 0.39 | 0.39 |
|  | Tebuthiuron | 3 | 3 |
| *H. uninervis* | Diuron | 0.34 | 0.34 |
|  | Atrazine | 3.5 | 3.5 |
|  | Hexazinone | 1.4 | 0.37 |
|  | Tebuthiuron | 4.6 | 4.6 |
