# Supplementary material for: Phytotoxicity of Four Photosystem II Herbicides to Tropical Seagrasses
Source: PLoS One. 2013 Sep 30;8(9):e75798. doi: 10.1371/journal.pone.0075798 (PMC3786934; doi:10.1371/journal.pone.0075798)
Supplement: Table S4 — Australian guidelines trigger values for ecological protection. (DOCX) [file pone.0075798.s004.docx]

**Table S4. Australian guidelines trigger values for ecological protection.**

| **Species protection** | **Diuron** | **Atrazine** | **Hexazinone** | **Tebuthiuron** | **Reference** |
| --- | --- | --- | --- | --- | --- |
| 90% | 2.3 | 2.5 | NA | 20* | [[52](#_ENREF_52)] |
| 95% | 1.6 | 1.4 | NA | 2* | [[52](#_ENREF_52)] |
| 99% | 0.9 | 0.6 | 1.2* | 0.02* | [[52](#_ENREF_52)] |
| 95% | NA | 13 | NA | 2.2 | [70] |
| 99% | NA | 0.7 | NA | 0.2 | [70] |
| Low reliability* | 0.2* | NA | 75* | 0.02* | [70] |

*Low reliability guidelines derived from data of fewer than five different species of at least four taxonomic groups. NA = not available.

^70^ ANZECC, ARMCANZ (2000) Australian and New Zealand guidelines for fresh and marine water quality. Australian and New Zealand Environment and Conservation Council and Agriculture and Resource Management Council of Australia and New Zealand. Available: http://www.environment.gov.au/water/publications/quality/nwqms-guidelines-4-vol1.html. Accessed 11 September 2013.
